# Simultaneous Transventricular-Orbitocranial Resection of Large Suprasellar Craniopharyngioma as Inspired by Jackson's Maneuver from 1863

**DOI:** 10.7759/cureus.517

**Published:** 2016-03-03

**Authors:** Walter C Jean, Hasan R Syed, Daniel R Felbaum, Joshua E Ryan, Amjad Anaizi

**Affiliations:** 1 Neurosurgery, Medstar Georgetown University Hospital

**Keywords:** skull base, endoscope, hybrid approach, microsurgery, craniopharyngioma

## Abstract

Traditional skull base techniques utilizing the microscope have allowed surgeons improved safe access to deep-seated lesions. More recent technical advances with the endoscope have allowed improved visibility and access to these previously difficult-to-reach regions. Most current literature emphasizes one technique over the other. We present a unique hybrid-type approach that tackles this not-infrequent surgical dilemma. This hybrid-type surgery resulted in a new technique that is a confluence of both open microsurgery and skull base corridors with an endoscope. Furthermore, a combined ventriculoscope approach adds extended assistance with resection. We detail the utility of this technique.

A patient presented with a large suprasellar lesion that was suspicious for a craniopharyngioma. Given improved survival with extent of resection, the goal of surgical intervention was maximal safe resection. The location of the tumor would have involved certain morbidity with deliberate residual if a skull base approach or endoscope-based approach was employed independently. As a result, the patient underwent a hybrid-type operation using a multi-corridor split-surgical team approach for the resection of her tumor.

The patient underwent hybrid surgery via a combined open microsurgical craniotomy, endoscopic resection, and a ventriculoscope-assisted approach. The ventriculoscope access allowed for resection of the intraventricular portion of the tumor and guided the extent of resection from the microsurgical corridor. Additionally, from a separate craniotomy, the suprasellar component was resected using both standard skull base and endoscope-assisted techniques. The patient tolerated the procedure well without additional morbidity provided from the multi-corridor hybrid technique.

The hybrid surgery resulted in a new multi-modality, split-surgical team approach providing maximal visualization with minimal added morbidity to resect a lesion difficult to access. This hybrid technique may be an effective piece of the surgeon’s armamentarium to provide improved patient outcomes.

## Introduction

On May 2, 1863, the Army of the Potomac was on the south shore of the Rappahannock river, facing off against Robert E. Lee’s Army of Northern Virginia. The left flank of the Federals was protected by the river, but the right flank was “up in the air,” unprotected by any natural geographic elements. Lee split his out-numbered forces and sent Gen. Thomas “Stonewall” Jackson’s forces on a march around the Federals right flank. His subsequent attack completely unhinged the unprotected Federal forces and made them collapse towards Lee’s main forces near Chancellorville. The maneuver was one of the most inspired in military history and the result was a resounding Confederate victory.

When a large suprasellar tumor invades the third ventricle, it also has an unprotected “flank.” It is anchored at the suprasellar region, but is unattached in the ventricle. A unidirectional approach to remove the tumor through the ventricle is suboptimal, as the optic chiasm is on the far end of the tumor. This provides a line of approach that is far from the surgeon and is difficult to visualize and protect critical neural structures. Alternatively, a unidirectional approach for the same tumor from the anterolateral pterional corridor has a similar problem. The ventricular component would be hard to see making removal technically difficult. However, utilizing a split-team approach, one may be able to achieve optimal results. This can be performed with a main resecting team working through the conventional anterolateral corridor, and the other team using a ventriculoscope to deliver the ventricular component of the tumor towards the main resecting team. 

In this study, we describe the use of this “Jackson’s maneuver” in such a split-team approach to manage a large craniopharyngioma with ventricular extension. Using a combined endoscopic/microscopic resection technique and simultaneous ventriculoscope assistance, the tumor was successfully removed.

## Technical report

Case Description

**Figure 1:**
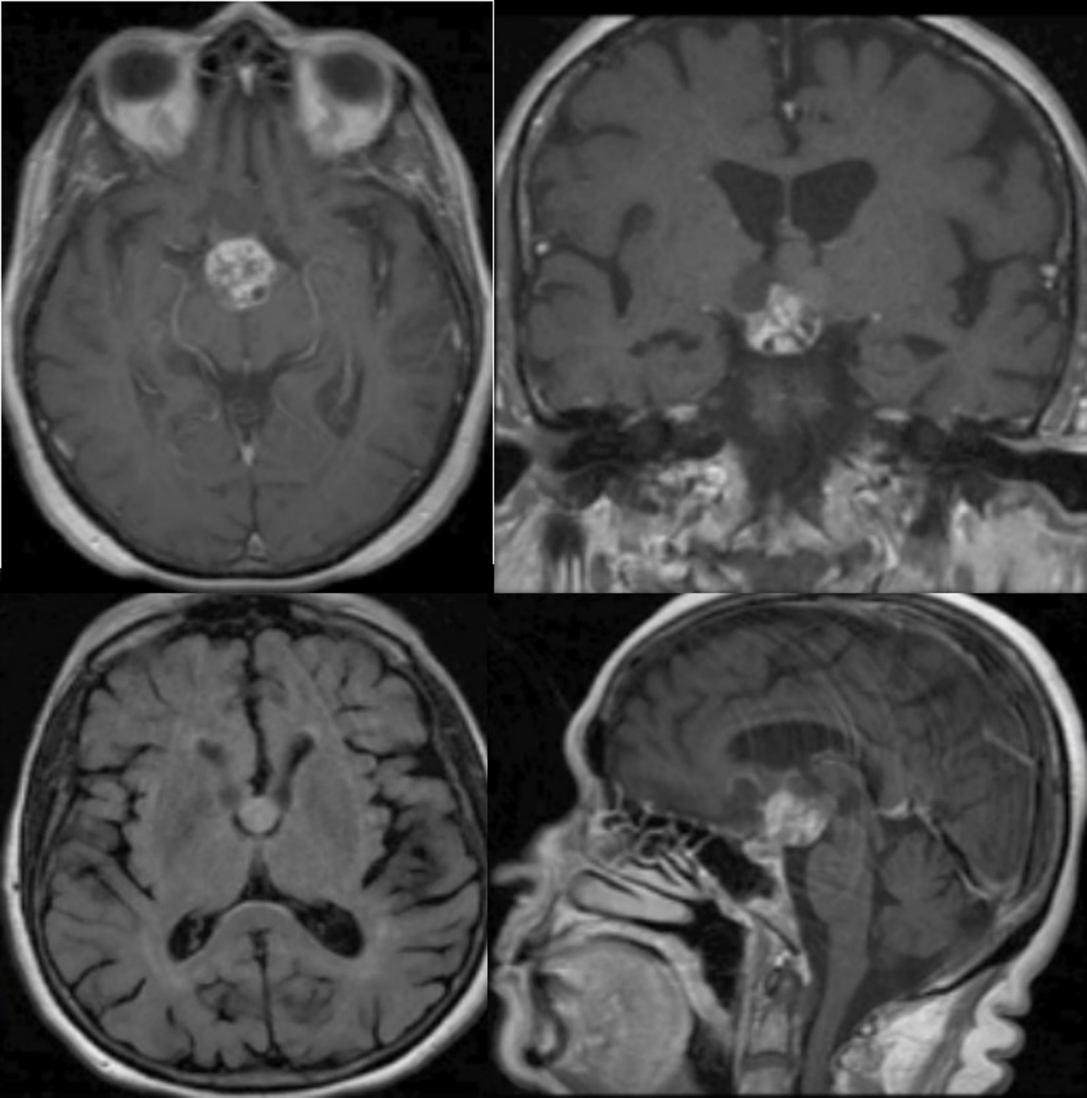
Preoperative MRI revealing a mass suspicious for craniopharyngioma Axial, coronal, and sagittal reconstructions of the patient's preoperative MRI. It depicts an enhancing lesion with a predominantly suprasellar component with third ventricular extension.

An 80-year-old woman presented with progressive visual deficits, memory loss, and urinary urgency. Imaging of her brain showed a large solid-cystic suprasellar mass, with multilobulated extension into the third ventricle (Figure 1).

**Figure 2:**
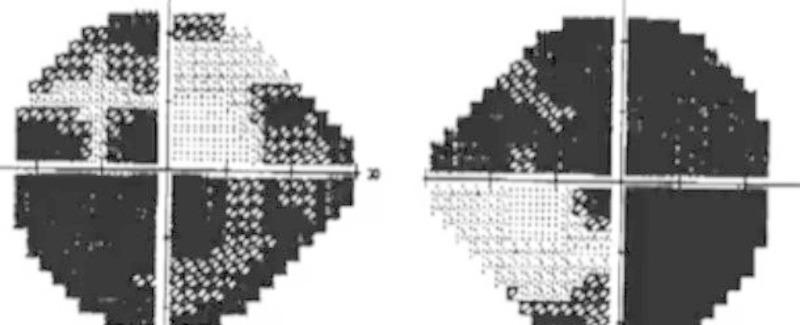
Formal Visual Fields Automated visual field testing of the patient revealing dark spots in almost all four quadrants of the patient's visual fields in both eyes. The dark spots are consistent with absence of vision.

Her formal visual field testing revealed extensive loss of vision (Figure 2).

Given radiographic and clinical progression of symptoms, surgery was offered due to her overall outstanding general health condition. Informed patient consent was obtained for this study.

Operative Description

**Figure 3:**
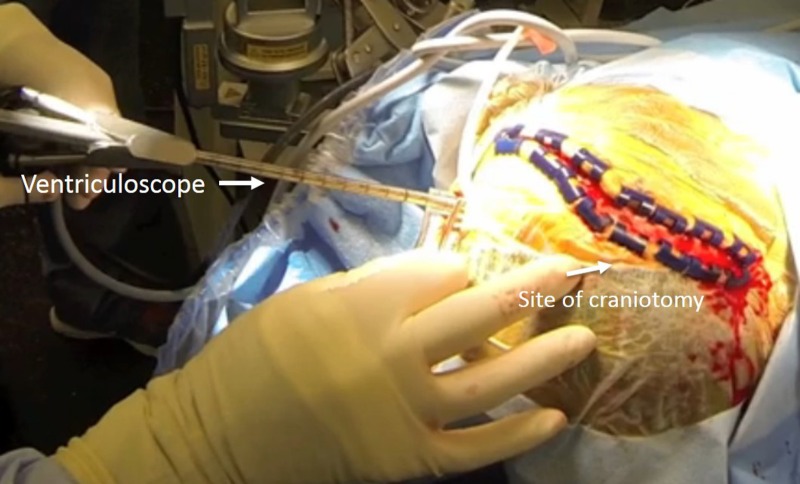
Intraoperative setup of hybrid operation The trajectory of the ventriculoscope is present in the intraoperative photo. The site of the future craniotomy is detailed on the right side of the picture.

At the start of the procedure, a ventricular puncture was made at the left Kocher’s point. A 14 French split trochar was introduced into the left lateral ventricular and followed by the ventriculoscope (Figure 3).

**Figure 4:**
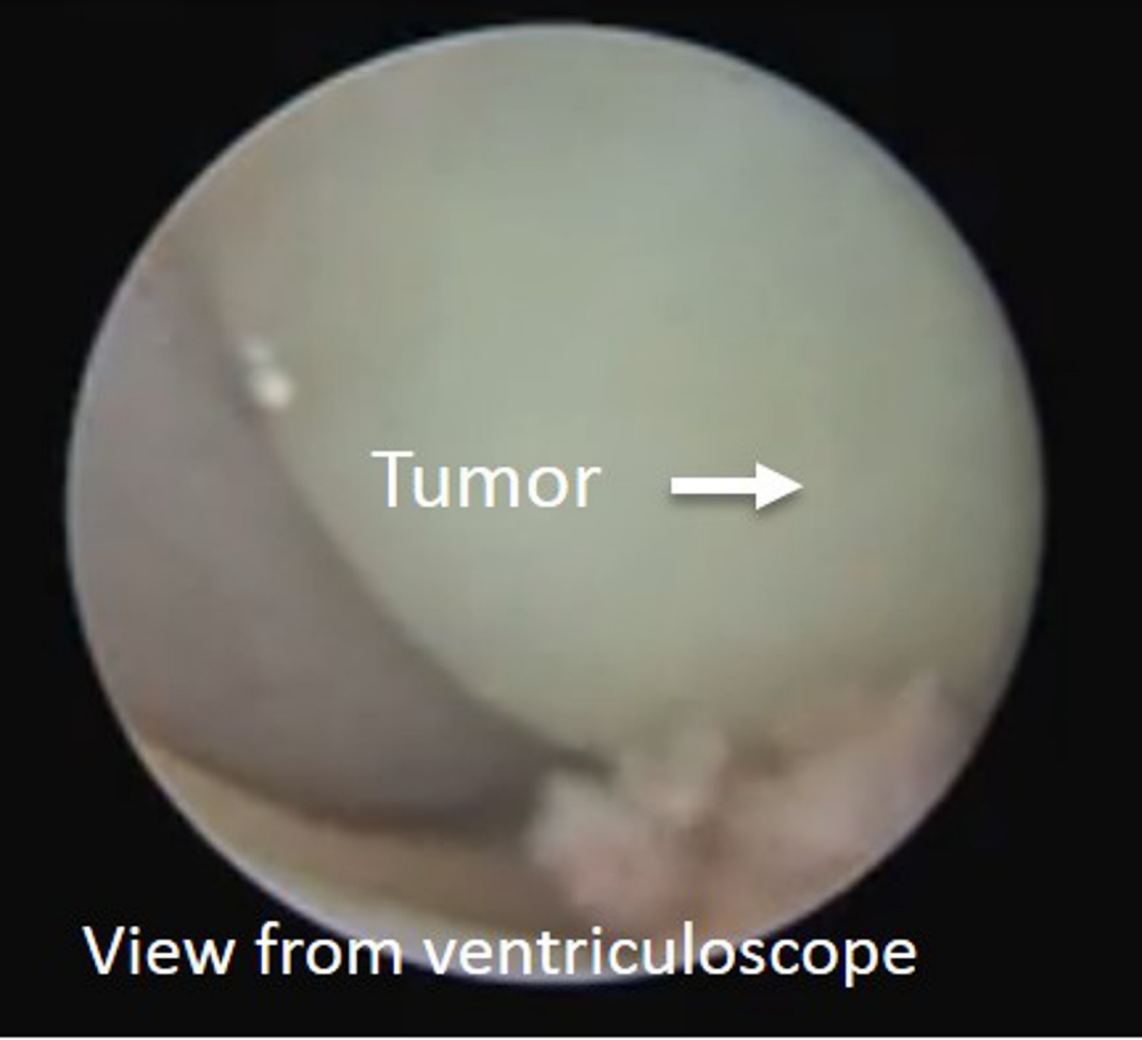
View from ventriculoscope Intraoperative view from the ventriculoscope. The third ventricular component of the tumor is present in the center of the picture.

Inspection with the ventriculoscope showed multiple cystic lobules of the tumor in the third ventricle consistent with the findings on preoperative imaging. The ventriculoscope was then removed pending further use in the procedure (Figure 4).

**Figure 5:**
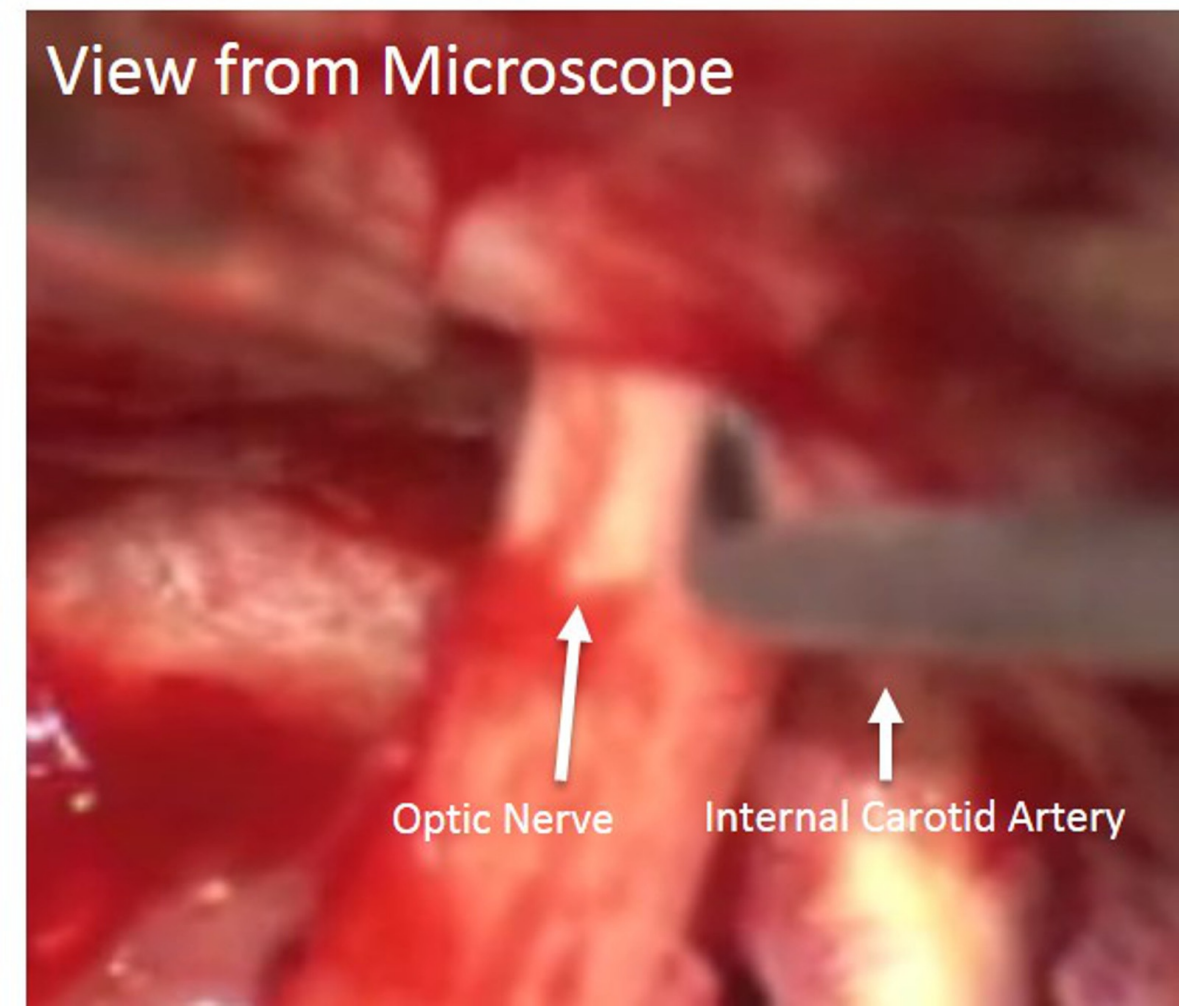
Microscopic view of regional anatomy Intraoperative view showing the release of the falciform ligament to liberalize the optic nerve. The opticocarotid window is seen as the access trajectory for the tumor.

The details of the one-piece orbitocranial craniotomy has been described elsewhere [1]. This was performed on the right side, followed by removal of the lateral portion of the orbital roof and opening of the optic foramen. The falciform ligament was incised intradurally. This allowed the right optic nerve to be completely released at the optic foramen, making it slightly less prone to stretch injury from subsequent manipulation (Figure 5).

**Figure 6:**
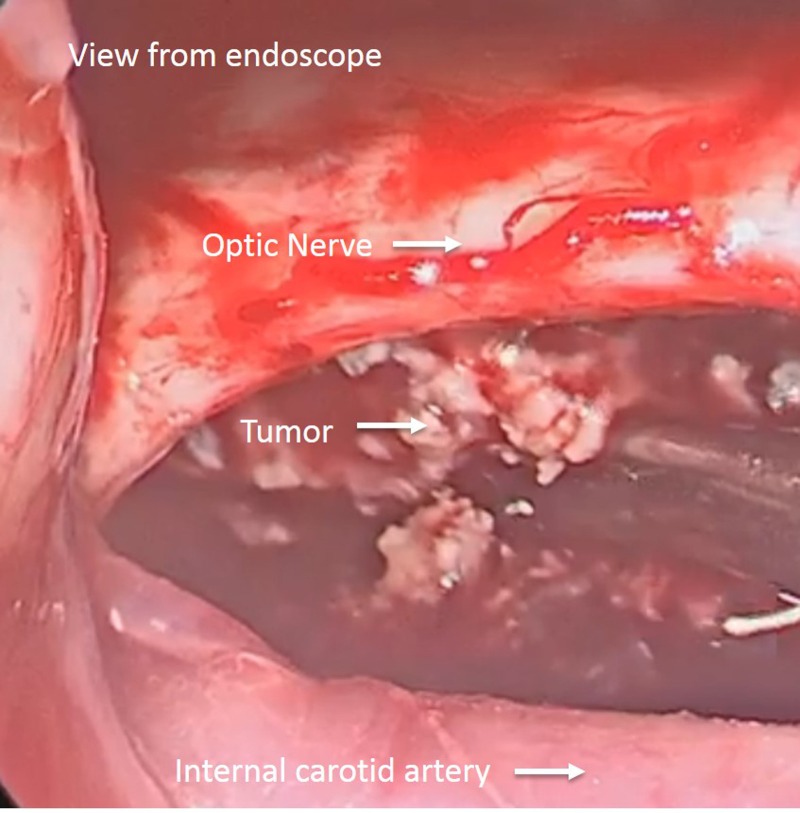
Endoscopic view of the tumor Endoscopic view showing access through the opticocarotid window. The optic nerve, internal carotid artery, and tumor are visualized from an angle not seen from the traditional microscopic approach.

Under microscopic visualization, the carotid arteries and optic nerves were freed from tumor and arachnoid adhesions on both sides. The tumor was then debulked, using the right opticocarotid window as well as the corridor between the two optic nerves. Once the limits of visualization were reached with the microscope, the endoscope was introduced through the craniotomy opening, from an anterolateral to posteromedial direction through the carotico-optic window. A combination of 0 and 30 degree endoscopes afforded panoramic views of the retrochiasmal portion of the tumor (Figure 6).

**Figure 7:**
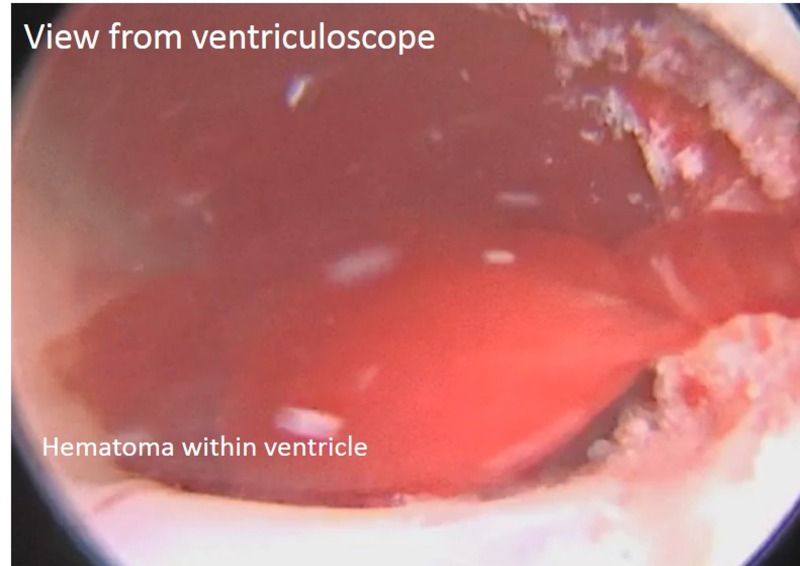
Ventriculoscopic view of hematoma View from the ventriculoscope depicting the obstructive hematoma.

A dissector, suction, and tumor grasper were used alternatively to remove the rest of the extraventricular tumor. Upon completion of the initial microsurgical resection, the simultaneous use of ventriculoscope and microscope was deployed. In the third ventricle, the cysts of the tumor were no longer present. However, a large blood clot was obstructing ventriculoscope investigation of potential tumor residual within the third ventricle (Figure 7).

**Figure 8:**
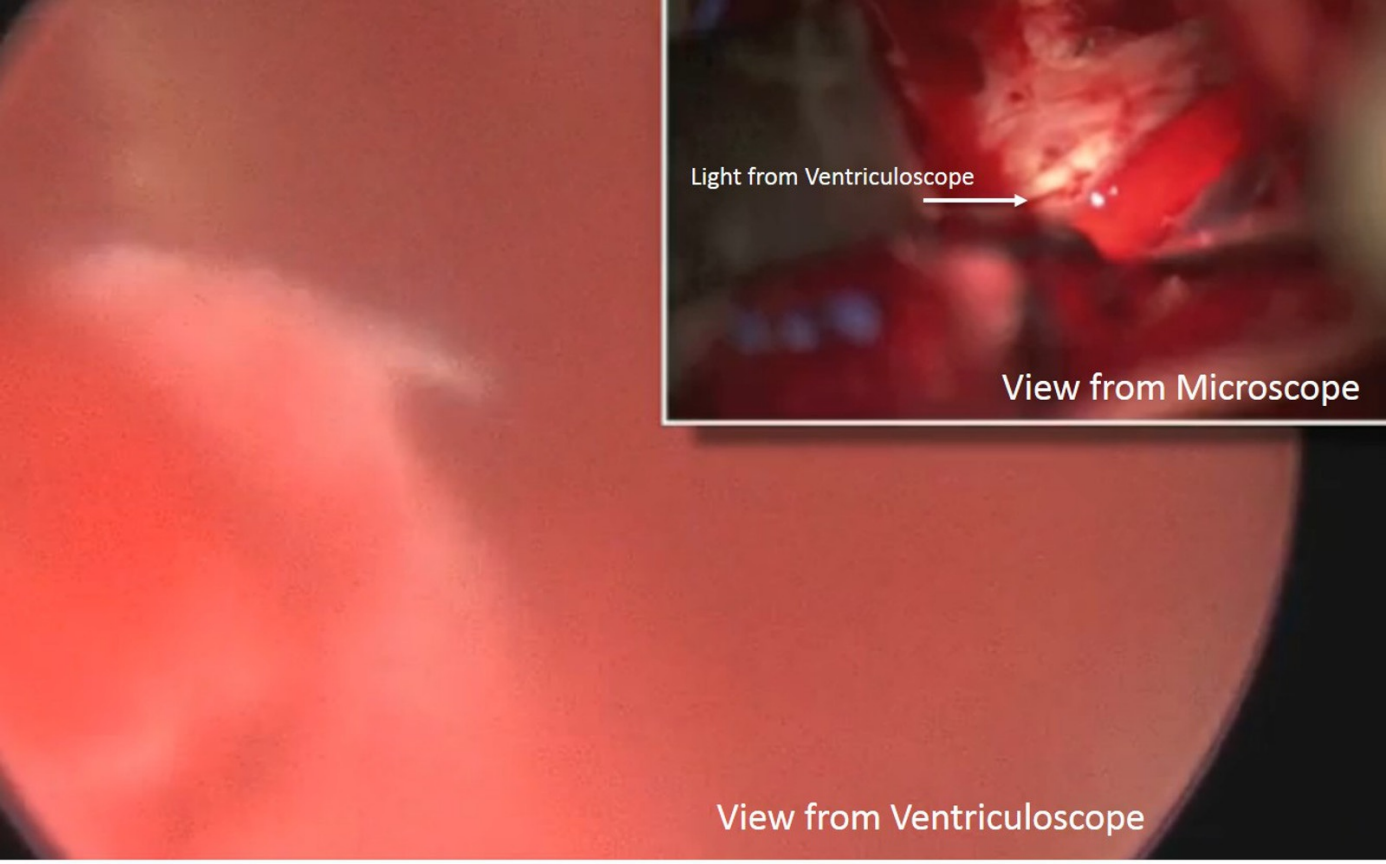
Combined view of ventriculoscope and microscope from separate trajectories The larger picture depicts the view from the ventriculoscope with hematoma present. From the smaller picture above, the microscope clearly sees the light from the endoscope. This provided to be a beacon to aid further surgical intervention.

Fortuitously, the microscope was able to clearly see the light from the ventriculoscope. The ability of the microscope to visualize the light emanating from the endoscope allowed the surgeon an opportunity to track it like an anatomical beacon behind the chiasm (Figure 8).

Using copious irrigation from the ventricular side, cyst content, operative debris, and the blood clot were delivered into the main resection field and removed conclusively. 

**Figure 9:**
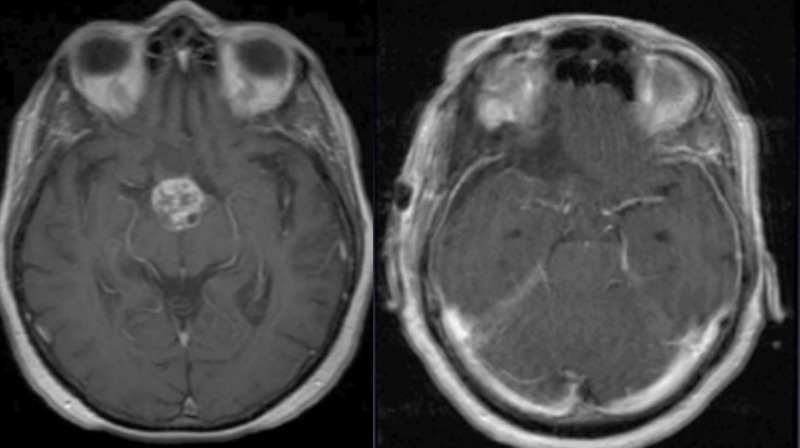
Comparison of preoperative and postoperative imaging The preoperative MRI is seen on the left, with the postoperative MRI on the right. The imaging comparison shows near complete total gross resection.

At the end, a curved probe was placed behind the chiasm into the third ventricle. The ventriculoscope was able to visualize this easier. This indicated that there was no longer any tumor between the two scopes, and the resection was complete. Intraoperative findings were confirmed with total gross resection on postoperative imaging (Figure 9).

The entirety of this surgical resection is detailed in an illustrative case video (Video 1)

**Video 1:** Illustrative video of patient presentation and operation

## Discussion

A “pincer movement” is a well-known military strategy in which a two-pronged attack confuses and entraps the enemy, leading to its defeat. Jackson’s maneuver in 1863 was especially brilliant because, by using a mobile force in a circumventing match to start the attack, the Confederates were able to dislodge the vulnerable flank of the Federals, driving them, in disorganized retreat, right into the main Confederate army lying in wait. The result was a massive defeat for the Federals.

A combined, two-directional attack on a suprasellar tumor has been described, but the few reports that exist all involve the use of the ventriculoscope to assist the extended endonasal resection for treating pituitary adenomas [2-9]. Our report concerns a craniopharyngioma that is entirely above the sella, and has normal pituitary function. In our view, the endonasal approach for this tumor would have been suboptimal because of a high risk of jeopardizing all her pituitary hormones [10]. It is for this reason that we chose the main resecting route of the anterolateral pterional corridor via a one-piece orbitocranial approach.

The resection of the main bulk of the tumor through the orbitocranial approach was done using a combined microscope-endoscope technique. Ichikawa et al. described a similar "hybrid” technique for craniopharyngioma surgery, but they used the endoscope mainly for inspection after resection was deemed complete [11]. In our case, the tumor projected dorso-posteriorly behind the optic chiasm, and since the chiasm was postfixed, visualization of the retrochiasmal component with the microscope was near impossible.

The ventriculoscope provided many functions in this procedure. At the start of the procedure, it provided information about the cystic nature of the tumor’s ventricular component. Although graspers and a 4Fr Fogarty balloon were readied to be used through the ventriculoscope, in the end, saline irrigation through the scope was sufficient to deliver both cystic content and resection-related blood clot into the main resection field.

Despite visualization that was partially compromised by resection debris late in the procedure, the light from the ventriculoscope was able to shine through into the main resection field. This allowed the microscope to track it, as it provided critical anatomic information about any residual tumor components. At the end of the procedure, the ventriculoscope provided unequivocal evidence that tumor removal from the third ventricle was complete.

Using the corridor provided by the orbitocranial approach, we placed the endoscope through the opticocarotid window, and gained superb visualization of the tumor’s retrochiasmic portion [1]. With a one-hand technique, alternating from a suction, a dissector, and tumor forceps, we were able to remove the most deep-lying portion of the tumor successfully, without damage to the optic chiasm and surrounding structures. In particular, we were able to attain views from a bottom looking up angle. This allowed even better visualization of residual tumor and allowed for a greater amount of resection.

Simultaneous bidirectional resections of tumors using multiple corridors have been nicknamed the “above-below” approach [6-7, 9]. We chose the moniker “Jackson’s maneuver” in honor of the brilliant Virginian strategist who inspired our own scheme.

## Conclusions

The "Jackson's maneuver" or multimodality split team approach is a potentially important marriage of available surgical techniques to provide improved visibility and resectability to difficult-to-access lesions with minimal added morbidity. Further experience with this technique may allow future surgeons the ability to aid treatment of these lesions that are difficult to access.
